# Electrochemical Evaluation on Apical Leakage of AH26 Sealer after Smear Layer Removal

**Published:** 2007-01-20

**Authors:** Seddigheh Khedmat, Mostafa Rezaefar

**Affiliations:** 1 *Department of Endodontics, Dental School, Tehran University of Medical Sciences, Tehran, Iran*; 2 *General Practitioner, Tehran, Iran*

**Keywords:** AH26, Electrochemical Technique, Smear Layer

## Abstract

**INTRODUCTION:** Recently, attention has been drawn to the influence of smear layer removal on apical seal, and the relation of root canal filling material with canal wall surface in the presence and absence of the smear layer. The purpose of this study was to evaluate the influence of the smear layer removal on the apical sealing ability of AH26 sealer.

**MATERIALS AND METHODS:** Forty extracted human anterior teeth were used in this study. All teeth were decoronated at CEJ. Root canals were prepared; and before obturation, they were randomly divided into two groups (n=17): Group A in which the smear layer was left intact, and in Group B smear layer was removed. Six roots were served as controls. After obturation, microleakage was measured by the electrochemical method for 30 days with 3-day intervals. Data were then analyzed by using Mann-Whitney test.

**RESULTS:** Based on the results of this study, in the absence of the smear layer, a highly significant decrease of apical leakage was found (P<0.001).

**CONCLUSION:** This study showed that the removal of the smear layer can significantly improve the apical sealing ability of AH26 sealer.

## INTRODUCTION

Smear layer is a thin layer of smeared material with 1 to 2µm of thickness on the surface of the canal walls which is produced as a direct result of mechanical instrumentation. Smeared material is also packed into dentinal tubules for distances up to 40µm (1-2). As this layer, which covers the surface of the canal walls, might contain necrotic tissue and bacterial remnants within it, and its presence might prevent the penetration of intra canal medicaments into the dentinal tubules, removal of smear layer is assumed to be beneficial. In addition, a suitable surface must be prepared to allow intimate contact and adhesion within the root canal; thus, when the smear layer is removed, the patent tubules and clean walls may provide a better seal by allowing greater penetration of filling material into the dentinal tubules (3). Smear layer has organic and inorganic components. The use of sodium hypochlorite during and after instrumentation, final flush with EDTA solution followed by NaOCl was found to be most effective in removing superficial debris and smear layer from root canal walls. Crumpton *et al.* demonstrated that an efficient removal of the smear layer was accomplished with a final rinse of 1ml of 17% EDTA for 1 min followed by 3 ml of 5.25% NaOCl (4). Recently, attention has been drawn to the influence of smear layer removal on apical seal and the contact of root canal filling material with the surface of canal wall in presence and absence of the smear layer.

**Figure 1:**
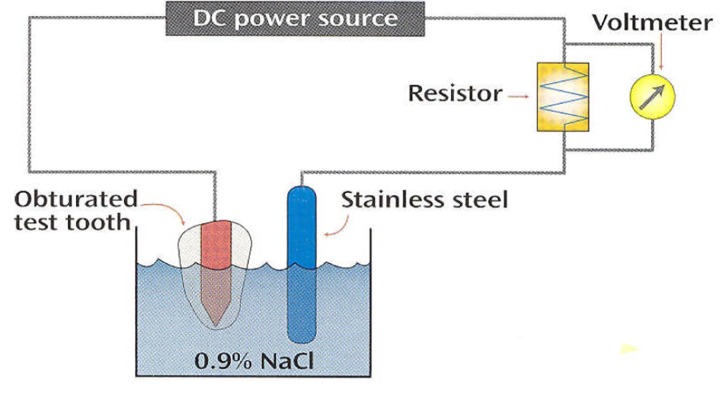
Schematic feature of digital galvometer

Electrochemical method is a quantitative technique for measuring of apical leakage. This method is based on the principle that electricity flows between two pieces of metal when both are immersed in an electrolyte and are connected to each other via an external power source. A galvanic current in this system will be detected only when the root canal leaks (Figure 1). Economides *et al.,* by the electro-chemical method, found a statistically significant reduction of microleakage values after the smear layer removal in the group obturated with AH26. Removing the smear layer, however, had no significant effect on the sealing ability of Roth 811 (5). In another electrochemical study, significant reduction of apical leakage was reported after removal of smear layer prior to canal obturation by ultrafil system (6). Others showed that the presence or absence of smear layer had no significant effect on the apical seal of injected thermoplasticized gutta-percha or lateral condensation with or without sealer (7). White *et al.*, In a SEM study, showed that the presence of smear layer prevents the entrance of filling materials into the dentinal tubules. However, some plastic root canal materials and sealers entered the dentinal tubules when the smear layer was removed prior to filling (8).

The purpose of this study was to evaluate the influence of the smear layer on the apical sealing ability of AH26 sealer by an electrochemical test method.

## MATERIALS AND METHODS

Forty extracted human anterior teeth with straight canals were used. Teeth were immersed in a 2.6% NaOCl for 20min to remove soft tissue from root surfaces. After cleaning, they were rinsed and stored in a normal saline solution. All teeth were decoronated at CEJ using high-speed handpiece with a cooling system.

A K-file #15 was passed through the apex and the working length was established at 1mm beyond the apex. The apical portion of all root canals were enlarged to #35, and rest of the canal length was flared to #70 by circumferential filing using step-back technique. Each root canal was irrigated with 2ml of 0.5% NaOCl solution after instrumentation with each file.

Upon completion of instrumentation, teeth were randomly divided into two groups (n=17): Group A- teeth received no further treatment and the smear layer remained attached to dentin and Group B- the smear layer was removed as follows: The canals were irrigated with 1ml of 17% EDTA for 1min followed by 3ml of 5.25% NaOCl. Then all roots in two groups were obturated by lateral condensation method using gutta-percha and AH26 sealer. Obturation accuracy was checked by two radiograph taken mesiodistally and buccolingually and it was considered sufficient when no voids were observed. Six roots were served as controls (3 roots as positive and 3 roots as negative). In the positive control group, teeth were obturated by lateral condensation without sealer and in negative control, teeth were left unfilled and root surfaces were covered completely with double layers of fingernail polish.

All roots were maintained in an incubator for 48h (100% humidity at 37C) for setting. A Gates-Glidden drill was used to remove the coronal portion of gutta-percha to 5-mm length. Root surfaces of all teeth were then covered with double layers of fingernail polish except 2mm of apical root portion.

A 15cm insulated wire was used for each root. 1cm of insulation coat was stripped off at each end of the wire. One end was placed into the coronal portion of each root canal until contact was made with the remaining gutta-percha and contact with gutta-percha was verified radiographically. Red sticky wax was adapted around the wire at the point of its entrance to immobilize the wire. The other end was placed in electrolyte (0.9% sodium chloride). A potential difference of 10V was applied across the system. A galvanic current in this system will be detected only when the root canal leaks. A continues electrolyte path has thus become established and can be measured by a sensitive galvanometer. The galvanic current was recorded in microampere as a voltage drop across the 100 Ohm standard resistance by the digital galvanometer. Data were recorded at 3-day intervals for 30 days. Statistical analysis of data was performed using Mann-Whitney test.

**Table 1:** Apical microleakage value (μA) in group

**Days**	**3**	**6**	**9**	**12**	**15**	**18**	**21**	**24**	**27**	**30**
#1	2.5	3	3	3	4.5	8.5	12.5	16.5	18	19.5
#2	0.5	2	2	2	2.5	2.5	2.5	3	3	3
#3	3.5	4	4.5	5	5	4	3	2.5	3	3.5
#4	1.5	2	2	2	2.5	2.5	3	3.5	4	5
#5	1	1.5	1.5	1.5	2	2	2.5	2.5	2.5	3
#6	1.5	1.5	1.5	2	2	2	2	2.5	3	3
#7	28	30	30	30	31	31	30.5	29.5	29.5	30
#8	2	2	2	2	2	2	3	3.5	4	4.5
#9	1	1.5	1.5	1.5	2	2	2.5	3	3.5	4
#10	1	1.5	2	2	2.5	2.5	3	2.5	2.5	3
#11	2	1.5	2	2	3	3	3	3	3	3.5
#12	10.5	10.5	11	13	14	14	13.5	13.5	14	15
#13	1.5	2.5	2.5	3.5	5.5	6	5.5	6	6.5	7
#14	1	1.5	1.5	2	2	2	2.5	3	3	3.5
#15	2.5	2.5	2.5	2.5	3	4.5	7	11.5	11	13
#16	2	2.5	3	3.5	3.5	3	4	4.5	5	5
#17	2	3	3.5	3.5	4	4	4.5	5	5	5.5

**Table 2:** Apical microleakage value (μA) in group B

**Days**	**3**	**6**	**9**	**12**	**15**	**18**	**21**	**24**	**27**	**30**
#1	0.5	0.5	0.5	1	1	1.5	1	1.5	1.5	2
#2		0	0.5	0.5	0.5	0.5	0.5	1	1	1
#3	0	0.5	0.5	0.5	0.5	0.5	0.5	0.5	0.5	0.5
#4	1	1	1	1	1	1	1	1	1	1
#5	0	0	0	0	0	0.5	0.5	0.5	0.5	0.5
#6	0	0	0	0.5	0.5	0.5	0.5	0.5	0.5	1
#7	0	0	0.5	0.5	0.5	0.5	1	0.5	1	1.5
#8	1	1	1	1	1.5	1.5	1.5	1.5	1.5	1.5
#9	0	0	0	0	0	0	0	0	0.5	0.5
#10	0.5	0.5	0.5	1	1	1	1	1.5	1.5	1.5
#11	0	0	0	0	0	0	0	0	0	0
#12	0	0	0	0.5	0.5	0.5	0.5	1	1	1
#13	0	0	0	0	0	0.5	0.5	0.5	1	1
#14	0	1	1	1	1.5	1.5	1.5	1.5	2	2.5
#15	0.5	0.5	0.5	1	1	1	1	1.5	1.5	1.5
#16	0	0	0	0	0	0	0	0	0	0
#17	0	0	0	0	0.5	0.5	0.5	0.5	1	1

## RESULTS

Most of the specimens in two groups showed low initial leakage that increased during the test period (Table 1),(Table 2). The mean microleakage values (Current flow) and standard deviation (SD) of two groups in relation to the observation periods are given in Table 3. No leakage was observed in negative controls during the experiment which indicated that two layers of fingernail polish were effective means of preventing electrolyte penetration. Microleakage in positive controls was higher than others. Comparison of the mean microleakage values in two groups showed that in group A the micro leakage was higher than group B in all observation periods.

Data analysis by using Mann-Whitney test indicated a highly significant difference between two groups in all periods of observation (P<0.001).

## DISCUSSION

The advantage of the electrochemical technique for testing leakage is that it can be monitored continuously during the test while number of leaking teeth and the degree of leakage can be determined.

The technique has clear advantage over dye-penetration and radioisotope techniques which are leak/non leak methods and do not permit monitoring of the specimens on a continuous basis (9).

This method allows the measurement and comparison of leakage values in the same teeth during observation periods. Besides, it is a reliable and sensitive method since the cl^-^ used as tracer is 1.98 A^°^ in diameter, whereas bacteria, such as lactobacillus and streptococcus, are 2 and 0.5, respectively (10). Electrochemical data in present study showed a highly significant decrease for apical leakage in the absence of the smear layer (P<0.001).

This is in agreement with results of Kennedy *et al. *who found that smear layer removal can improve the sealing of root canals (11). Evans and Simon studied the effect of the smear layer on teeth obturated with lateral condensation or thermoplasticized injection of gutta-percha. The removal of smear layer had no effect on micro leakage value in either technique (7). The effect of the smear layer on root canals obturated with the ultrafil system has been studied. It has been concluded that the presence or absence of a smear layer did not influence the sealing ability, but the presence of smear layer increased the microleakage when sealer was used (6).

**Table 3:** Mean apical microleakage values (μA) of the tested groups in relation to observation periods

**Days**	**A(Smear layer+)**	**B(Smear layer-)**	**P-Valve**
	**Mean ± SD**	**Mean± SD**	
3	2.294±3.5490	0.206±0.3561	<0.001
6	2.676±4.1077	0.294±0.3976	<0.001
9	3.059±4.2238	0.353±0.3859	<0.001
12	3.118±4.1365	0.500±0.4330	<0.001
15	3.324±4.3118	0.588±0.5073	<0.001
18	3.971±6.9028	0.676±0.4957	<0.001
21	4.794±6.9411	0.676±0.4682	<0.001
24	5.324±6.8464	0.794±0.5607	<0.001
27	5.500±6.9978	0.941±0.5557	<0.001
30	5.971±7.5673	1.059±0.6587	<0.001

This finding confirms that the effect of smear layer on root canal obturation is more correlated to the presence of the sealer than the primary filling material (Gutta-percha).

Economides *et al.* in their study showed that removal of smear layer improved the quality of apical sealing when AH26 sealer was used. On the contrary with Roth 811, no statistically significant difference was detected (5). Therefore, it seems that some kinds of sealers may respond differently in the absence of smear layer. Lester and Boyde have suggested that without smear layer, the particle size of the sealer should be less than the average diameter of the dentinal tubules (12).

In a SEM study White *et al.* observed that removal of smear layer enabled the filling material to enter the dentinal tubules (8).

Decrease of apical microleakage in smear- free canals in our study may be due to improved mechanical locking of AH26 sealer into patent tubules, better adhesion to cleaner canal walls and greater canal wall sealing surface area as smear layer is an interface between the filling material and the dentin wall.

## CONCLUSION

According to the results of present study, removal of the smear layer significantly improves the apical sealing ability of AH26 sealer; however further study is needed in order to prove this matter.
